# Dielectrophoresis-Based Positioning of Carbon Nanotubes for Wafer-Scale Fabrication of Carbon Nanotube Devices

**DOI:** 10.3390/mi12010012

**Published:** 2020-12-25

**Authors:** Joevonte Kimbrough, Lauren Williams, Qunying Yuan, Zhigang Xiao

**Affiliations:** 1 Department of Electrical Engineering and Computer Science, Alabama A&M University, Normal, AL 35762, USA; jkimbro3@bulldogs.aamu.edu (J.K.); lwilli81@bulldogs.aamu.edu (L.W.); 2 Department of Biological and Environmental Science, Alabama A&M University, Normal, AL 35762, USA; qunying.yuan@aamu.edu

**Keywords:** dielectrophoresis (DEP), semiconducting carbon nanotubes, carbon nanotube field-effect transistors (CNTFETs)

## Abstract

In this paper, we report the wafer-scale fabrication of carbon nanotube field-effect transistors (CNTFETs) with the dielectrophoresis (DEP) method. Semiconducting carbon nanotubes (CNTs) were positioned as the active channel material in the fabrication of carbon nanotube field-effect transistors (CNTFETs) with dielectrophoresis (DEP). The drain-source current (I_DS_) was measured as a function of the drain-source voltage (V_DS_) and gate-source voltage (V_GS_) from each CNTFET on the fabricated wafer. The I_DS_ on/off ratio was derived for each CNTFET. It was found that 87% of the fabricated CNTFETs was functional, and that among the functional CNTFETs, 30% of the CNTFETs had an I_DS_ on/off ratio larger than 20 while 70% of the CNTFETs had an I_DS_ on/off ratio lower than 20. The highest I_DS_ on/off ratio was about 490. The DEP-based positioning of carbon nanotubes is simple and effective, and the DEP-based device fabrication steps are compatible with Si technology processes and could lead to the wafer-scale fabrication of CNT electronic devices.

## 1. Introduction

Carbon nanotube-based electronic devices such as carbon nanotube field-effect transistors (CNTFETs) and electronic circuits have been investigated extensively in the past years for the application of future nanoelectronic devices [1,2,3,4,5,6,7,8,9,10,11,12,13,14,15]. Since carbon nanotubes (CNTs) have a high aspect (ratio of the length to diameter) with a diameter of several nanometers and a length of several micrometers, the electrons or holes in the tubes are subject to a strong quantum-confinement effect in all directions perpendicular to the tube, making their transport in the tube a ballistic transport with less scattering and collisions [16,17]. These properties are not available in bulk electronic materials and can make carbon nanotube field-effect transistors (CNTFETs) operate at a high speed and low power without energy dissipating in the tube [18].

The wafer-scale fabrication of carbon nanotube electronic circuits can lead to the production and application of carbon nanotube electronic devices and has recently been reported by researchers [19,20,21]. In this paper, we report the wafer-scale fabrication of carbon nanotube field-effect transistors (CNTFETs) using dielectrophoresis (DEP), which is simple and unique compared to other methods [22,23]. Carbon nanotubes were positioned and assembled as the active channel material in the wafer-scale fabrication of carbon nanotube field-effect transistors (CNTFETs) using electric field-directed dielectrophoresis (DEP). Dielectrophoresis (DEP) is a translational motion of neutral matter caused by the polarization effects in a nonuniform electric field and has recently been investigated theoretically and experimentally for the deposition and alignment of CNTs [24,25,26,27,28,29]. It is also effective in assembling other materials such as liquid metal [30]. Aligned and dense carbon nanotubes (CNTs) can be obtained in the DEP process by optimizing the ac frequency, the trapping time, and the CNT solution concentration [31,32,33]. Impurities such as the metal impurity in CNTs, which come from the growth of CNTs due to the metal catalyst, could electrically short CNTFETs and cannot be tolerated in the fabrication of CNTFETs [34,35]. However, the impurities can be filtered in the pre-DEP process with solvents such as N-methyl pyrrolidone (NMP), making the DEP-based fabrication of CNTFETs appealing for obtaining high-performance carbon nanotube field-effect transistors and electronic devices. Since the DEP-based device fabrication steps are compatible with Si technology processes, they are capable of being further optimized by following process development protocols practiced by the semiconductor industry and can lead to the wafer-scale fabrication of CNT electronic devices and sensors.

## 2. Experimental Details

### 2.1. CNT-NMP Solution Preparation

Carbon nanotube (CNT) powder with 98% semiconducting CNTs (from the NanoIntergris Company) was used in this research. The CNT powder was dispersed ultrasonically in the Nmethyl-2-pyrrolidone (NMP) solution (from the Sigma-Aldrich Company) for the dielectrophoresis (DEP) process. The CNTs were subjected to a cleaning process for filtering any possible impurity in the CNTs before being used for the DEP process. 1 mg CNT powder was added to 20 ml NMP and sonicated for 10 min. The CNT solution was then centrifuged at 14,000 rpm for 30 min. The resultant supernatant was decanted for the sedimented carbon nanotubes, which were again subjected to the cleaning cycle with a fresh NMP. The cleaning process was repeated three times. Finally, the sedimented carbon nanotubes were added into a 30 ml NMP solution; the solution was sonicated for 5 min. After the sonication, the carbon nanotubes were separated and uniformly dispersed in the NMP solution, and the CNT-NMP solution was ready for the dielectrophoresis process in the following device fabrication.

### 2.2. FET Device Fabrication

**Figure 1:**
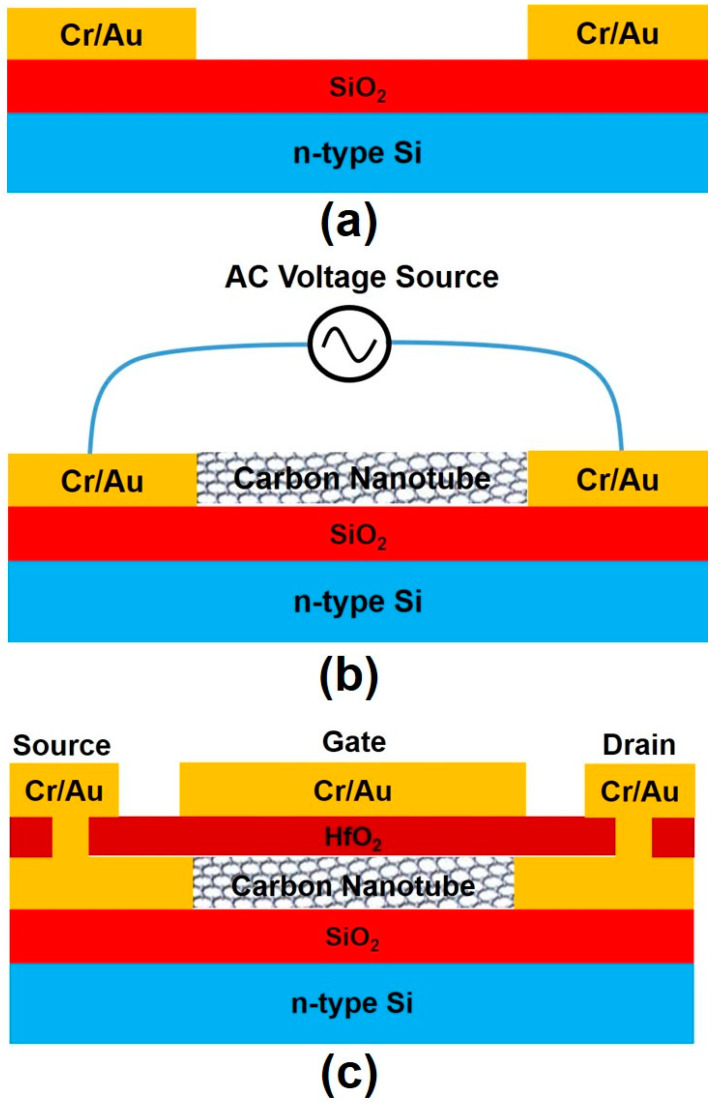
Carbon nanotube field-effect transistor (CNTFET) device fabrication steps. (**a**) Cr/Au electrode definition. (**b**) Alignment and assembling of CNTs with the DEP process under an applied sinusoidal voltage source between source and drain electrodes; the subsequent steps of the etching of Cr/Au films and the definition of HfO_2_ gate oxide are not depicted in this figure. (**c**) Source, drain, and gate metal electrode definitions.

**Figure 2:**
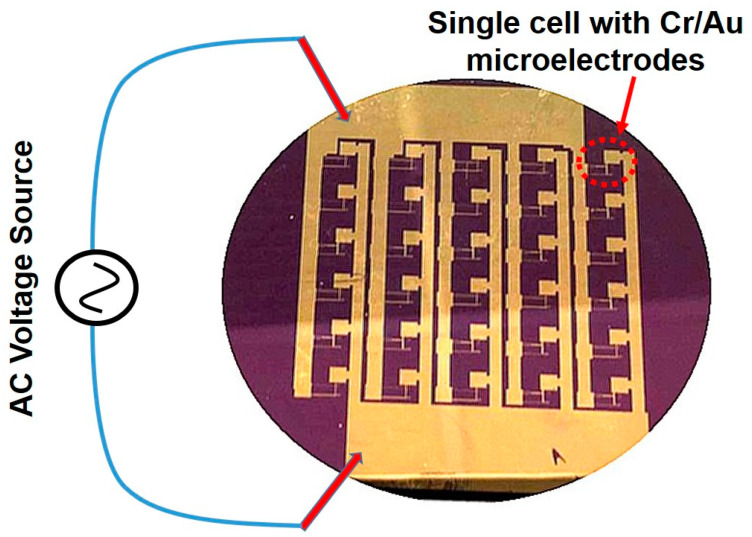
The wafer with 30 pairs of Cr/Au electrodes fabricated on it, where all the electrodes were connected in parallel to two major pads to which the ac voltages were applied during the subsequent DEP process.

A 3-inch diameter 350-µm thick n-type <100> silicon (Si) wafer (from Virginia Semiconductor, Inc.) was used as the substrate in this research. The Si wafer was about 350 µm thick and had a resistivity of about 3 Ω cm. The wafer was initially oxidized at 1100 °C for 120 min to grow a silicon dioxide (SiO_2_) layer of about 1 µm thickness using wet oxidation. A PVD 75 (Kurt J. Lesker Company, Pittsburgh, PA, USA) e-beam/thermal evaporation system was used to grow chromium (Cr)/gold (Au) thin films for the fabrication of Cr/Au electrodes and device metallization. The process chamber had a background pressure of 2 × 10^−7^ Torr, and the film thickness was controlled by an INFICON deposition monitor. An ALD-150LX system (Kurt J. Lesker Company, Pittsburgh, USA) was used to grow the hafnium dioxide (HfO_2_) thin film as the gate oxide in the fabrication of CNTFETs [36,37]. A set of four-piece photo masks was designed and used for the fabrication of the carbon nanotube field-effect transistors (CNTFETs). The photo masks were fabricated by Photoscience, Inc. Figure 1a–c show the fabrication steps of CNTFETs. The first mask was used to define and pattern 5-nm thick Cr/100-nm thick Au (99.99% pure from Alfa Aesar) source and drain electrodes with gaps of about 3 µm using standard ultraviolet (UV) photolithography and metal lift-off processes (Figure 1a). The thin Cr layer was used to enhance the adhesion of Au to the underlayer. The device wafer was designed to have 30 CNTFETs on it. After the first mask, 30 pairs of electrodes were fabricated on the wafer (Figure 2), and all the electrodes were connected in parallel to two major pads, which were applied with the ac voltages during the subsequent DEP process.

**Figure 3:**
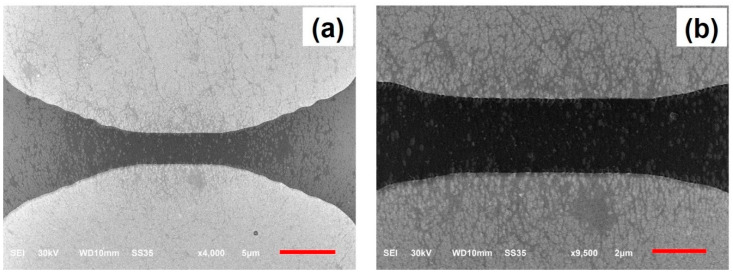
(**a**) SEM image of CNTs aligned and assembled between a pair of electrodes in a CNT-NMP solution with a higher concentration of CNTs using the DEP process; (**b**) enlarged view of the CNTs, showing a network-like profile of CNTs. The scale bar is 5 µm.

**Figure 4:**
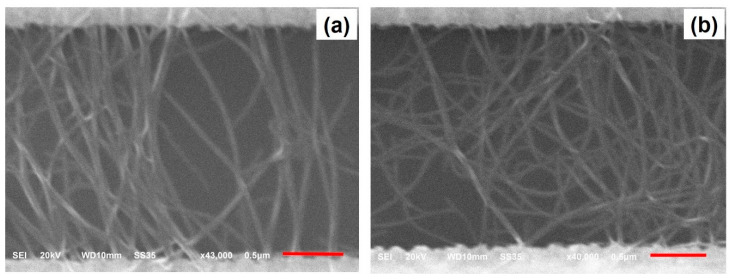
SEM images of CNTs aligned and assembled between the electrodes in two pairs of electrodes in a CNT-NMP solution with a lower concentration of CNTs using the DEP process, where both (**a**) and (**b**) show profiles of CNTs with some alignment of CNTs across the electrodes. The scale bar is 0.5 µm.

After the fabrication of the Cr/Au electrodes on the wafer, the dielectrophoresis (DEP) process was then performed to deposit and align CNTs across the gap of electrodes (Figure 1b). A sinusoidal voltage source was connected to the two major pads of the wafer (Figure 2), and the wafer was then dipped into a glass beaker filled with the CNT–NMP solution prepared as described above. A sinusoidal voltage with a peak-to-peak voltage of 10 V and frequency of 1 MHz was applied to the electrodes for 10 min. After that, the wafer was taken out from the beaker. The solvent was blown off the wafer surface with nitrogen gas. The wafer surface was then cleaned with 2-Propanol and rinsed in deionized (DI) water for 1 min. After that, the wafer was dried with nitrogen gas. The CNTs were imaged in a JEOL JSM-6610LV scanning electron microscope (JEOL Ltd, Akishima, Japan). Figure 3a shows the scanning electron micrograph (SEM) image of CNTs positioned between a pair of electrodes in a CNT-NMP solution with a higher concentration of CNTs using the DEP process, while Figure 3b is the enlarged view of the CNTs across the gap of electrodes, showing a network-like profile of CNTs. Figure 4a,b show the SEM images of CNTs positioned between the electrodes in a CNT-NMP solution with a lower concentration of CNTs using the DEP process for two pairs of electrodes. As shown in Figure 4a,b, although they were positioned with the same DEP process conditions, the CNTs in Figure 4a,b had varying profiles because of the random and uncontrollable property of CNTs dispersed in the CNT-NMP solution. Figure 4 also shows that the positioned CNTs are not single-tube and are bundling tubes (a single tube should have a diameter of several nanometers). The CNT-NMP solution with a lower concentration of CNTs was used for the final device fabrication.

The second mask was applied to create patterns for etching the Cr/Au metal film that connected with the pairs of electrodes using the UV lithography and to separate the electrodes into individual pairs of electrodes. After the UV lithography process, each pair of electrodes, together with the CNTs between the gap, was covered by Shipley 1818 positive photoresist, but all other areas on the wafer were open without being covered by the photoresist. The wafer was dipped in a gold etchant (Transene, Danvers, MA, USA) for 30 s, then in DI water for 1 min, and in a chromium etchant (Transene, Danvers, MA, USA) for 30 s to remove all the Cr/Au except that protected by the photoresist. The wafer was finally rinsed in DI water for 1 min and was dried with nitrogen gas. After that, a 10-nm thick HfO_2_ film was deposited over the whole wafer as the gate oxide using plasma-enhanced atomic layer deposition.

The third mask was applied to open a window through the HfO_2_ film on the source/drain for the final source/drain metal contact using the UV lithography. After the UV lithography process, the wafer was covered by Shipley 1818 photoresist, except for a 30 µm × 30 µm small area on the source/drain. The wafer was then dipped in buffered oxide etchant (BOE) (Transene, Danvers, MA, USA) for 3 min to etch the HfO_2_ through the patterned windows on the source/drain for the contact.

**Figure 5:**
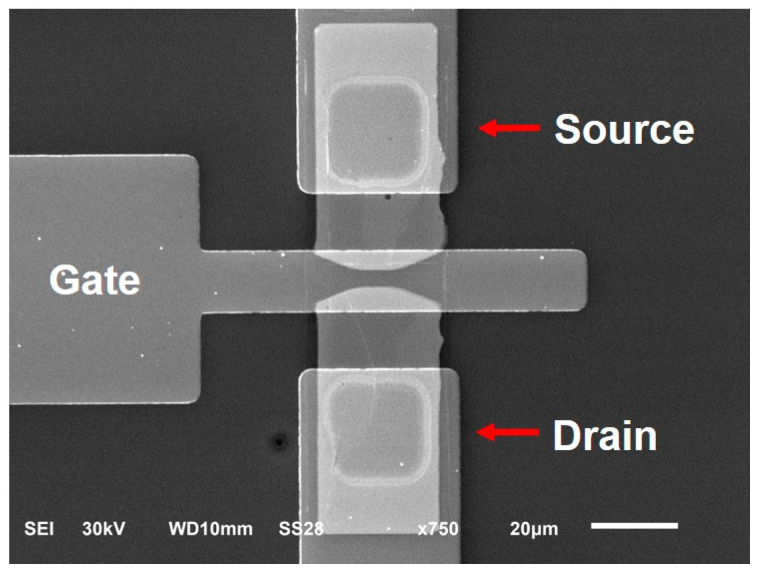
SEM image of a fabricated CNTFET.

Finally, the fourth photoresist mask aligned to the opened windows was applied to define the source, drain, and gate metal contacts (100-nm thick Au/5-nm thick Cr) via e-beam evaporation and metal lift-off. Again, Cr was used to enhance the adhesion of Au to the underneath layer (HfO_2_). Figure 1c shows the cross-sectional diagram of the CNTFET device, and Figure 5 shows the scanning electron micrograph of the top view of the fabricated CNTFET.

### 2.3. Electrical Measurements

Electrical contacts to the substrate, source, drain and gate electrodes were performed at room temperature using a probe station (CM-170 from Signatone, Gilroy, USA) equipped with four triax micromanipulated probes. The wafer substrate remained electrically unconnected (floating) during the measurements. Electrical signals for the device characterization were sourced and measured using a precision semiconductor parameter analyzer (4156C from Agilent, Santa Clara, CA, USA).

### 2.4. Electrical Breakdown Process

To increase the I_DS_ on/off ratios with the electrical breakdown method [38,39], CNTFETs were fabricated using the same processes. The IV curves and transfer characteristics of the CNTFETs were first measured as fabricated; after that, the devices were subjected to an electrical breakdown process, which consisted of sweeping V_DS_ from −8 V to 8 V at different V_GS_ values that varied from −8 V to 8 V in steps of 0.5 V until the I_DS_ values decreased to values that were about five to 10 times smaller. Then, the IV curves and transfer characteristics of the CNTFETs were measured again.

## 3. Results and Discussion

After completion of the device fabrication, each carbon nanotube field-effect transistor (CNTFET) fabricated on the wafer was probed and measured one by one as described above. It was found that 26 (87%) of the 30 CNTFETs on the wafer were functional, while the other four were broken. Among the 26 functional CNTFETs, 30% of the CNTFETs had a drain-source current (I_DS_) on/off ratio larger than 20 while 70% of the CNTFETs had a drain-source current (I_DS_) on/off ratio under 20. The highest drain-source current (I_DS_) on/off ratio was about 490. The reason why the electrical properties such as the I_DS_ on/off ratio varied in the CNTFETs was because the profile and number of CNTs positioned between the electrodes varied in the CNTFETs, as shown in Figure 4a,b. Both the profile and number of aligned CNTs can have effects on the electrical property of CNTFETs. The CNTFET with more CNTs had a lower channel resistance and therefore a higher I_DS_ value. The carbon nanotubes used in this research had about 98% semiconducting carbon tubes and about 2% metallic carbon tubes. The metallic tubes in the CNTs could be the major factor responsible for the variation of the I_DS_ on/off ratio in the CNTFETs. The metallic carbon nanotube has a zero band gap and functions like metal, and the resistance cannot be modulated with the applied gate voltage to CNTFETs. The semiconducting carbon nanotube contributes to the increase of the I_DS_ on/off ratio in a CNTFET, while the metallic carbon nanotube functions in an opposite way [40,41]. A higher number of metallic CNTs in the channel of CNTFET makes the modulation of channel resistance more difficult, resulting in a lower I_DS_ on/off ratio. Since the metallic carbon nanotubes could not be filtered in the DEP process, they were randomly assembled into the channel of CNTFET, resulting in the variation of the I_DS_ on/off ratio in CNTFETs.

**Figure 6:**
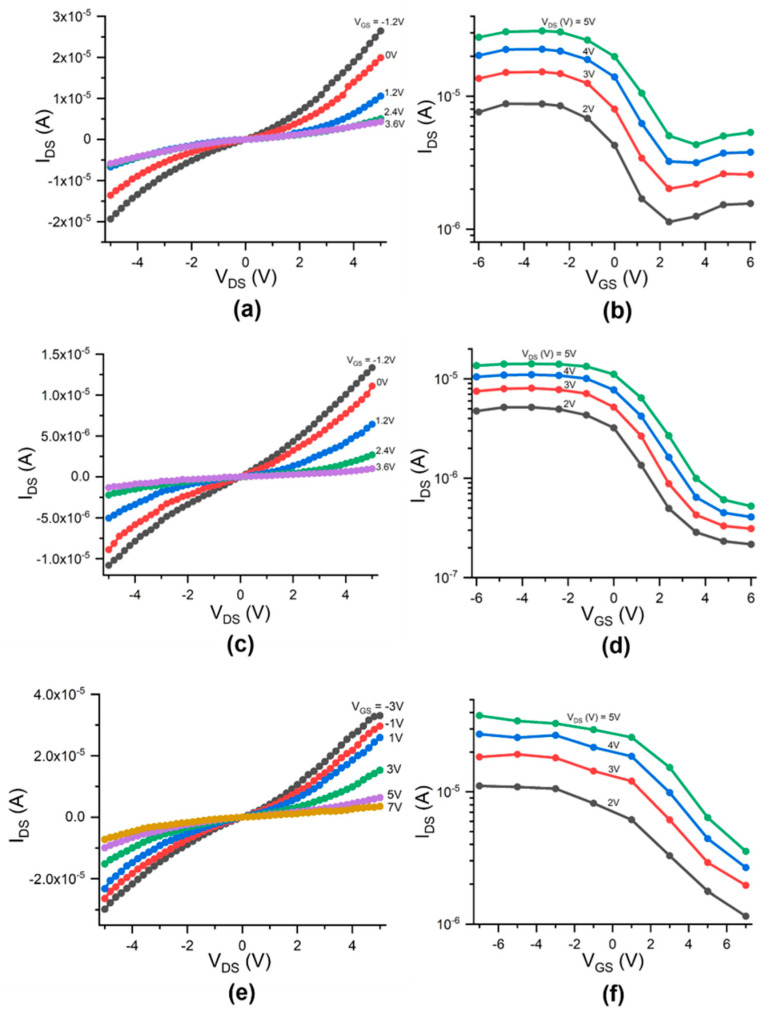
Electrical measurement results for three functional CNTFET devices: (**a**) Drain-source current (I_DS_) versus drain-source voltage (V_DS_) and gate voltage (V_GS_) and (**b**) transfer characteristics at V_DS_ = +5 V, +4 V, +3 V, and +2 V for device one, where the I_DS_ on/off ratio is 8; (**c**) Drain-source current (I_DS_) versus drain-source voltage (V_DS_) and gate voltage (V_GS_) and (**d**) transfer characteristics for device two, where the I_DS_ on/off ratio is 27; (**e**) Drain-source current (I_DS_) versus drain-source voltage (V_DS_) and gate voltage (V_GS_) and (**f**) transfer characteristics for device three, where the I_DS_ on/off ratio is 10.

Figure 6a–f show the drain-source current (I_DS_) versus the drain-source voltage (V_DS_) at various V_GS_ values (Figure 6a,c,e) and the I_DS_-V_GS_ transfer curves of the CNTFET at various V_DS_ values (Figure 6b,d,f) for three functional devices, respectively. The I_DS_ on/off ratio, defined as the maximum I_DS_ value divided by the lowest one in the I_DS_-V_GS_ transfer curve, is 8 for the device in Figure 6b, 27 for the device in Figure 6d, and 10 for the device in Figure 6f. All the devices present electrical properties of a p-channel field-effect transistor (FET), which are like those of the p-type carbon nanotube transistors reported by other researchers [42,43]. The I_DS_ is sensitive to the V_GS_ and can be modulated by V_GS_, as shown in the figure. When increasing V_GS_ negatively, the I_DS_ values increase correspondingly. P-type transistors conduct holes when a negative voltage is applied to the gate. They do not conduct electrons, even at high positive gate voltages. The physical explanation for this is that the Fermi level at the contact metal-nanotube junction is closer to the valence band of the nanotube, leading to hole conduction and p-type behavior. The holes see a small barrier and can thus tunnel through, whereas the electrons see a much larger barrier and are not able to tunnel [44].

**Figure 7:**
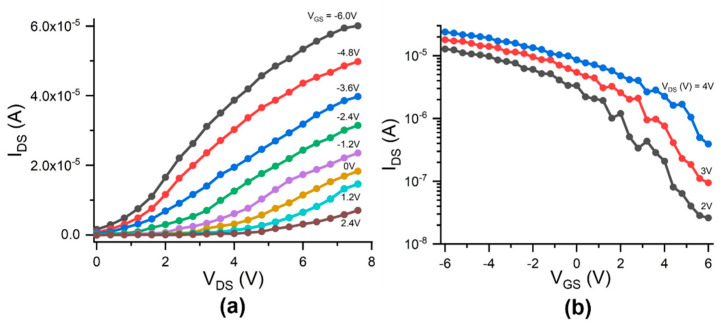
(**a**) Drain-source current (I_DS_) versus drain-source voltage (V_DS_) and gate voltage (V_GS_) and (**b**) transfer characteristics at V_DS_ = 5 V, 4 V, 3 V, and 2 V for a functional CNTFET, respectively; the I_DS_ on/off ratio is 490.

Figure 7a shows the drain-source current (I_DS_) versus the drain-source voltage (V_DS_) at various V_GS_ values for the functional CNTFET that had the highest I_DS_ on/off ratio among all the functional devices on the fabricated wafer, and it presents better IV curves, with the saturation like that of a silicon-based p-channel field-effect transistor [45]. Figure 7b shows the I_DS_-V_GS_ transfer curves of the CNTFET at V_DS_ = 4 V, 3 V, and 2 V. The I_DS_ on/off ratio is 61 at V_DS_ = 4 V, 190 at V_DS_ = 3 V, and 490 at V_DS_ = 2 V. As described above, the reason why the CNTFET had a higher I_DS_ on/off ratio is possibly because less or no metallic tubes were assembled in the channel during the DEP process. The I_DS_-V_DS_ curves in Figure 7a are comparable with those reported by other researchers [46,47].

**Figure 8:**
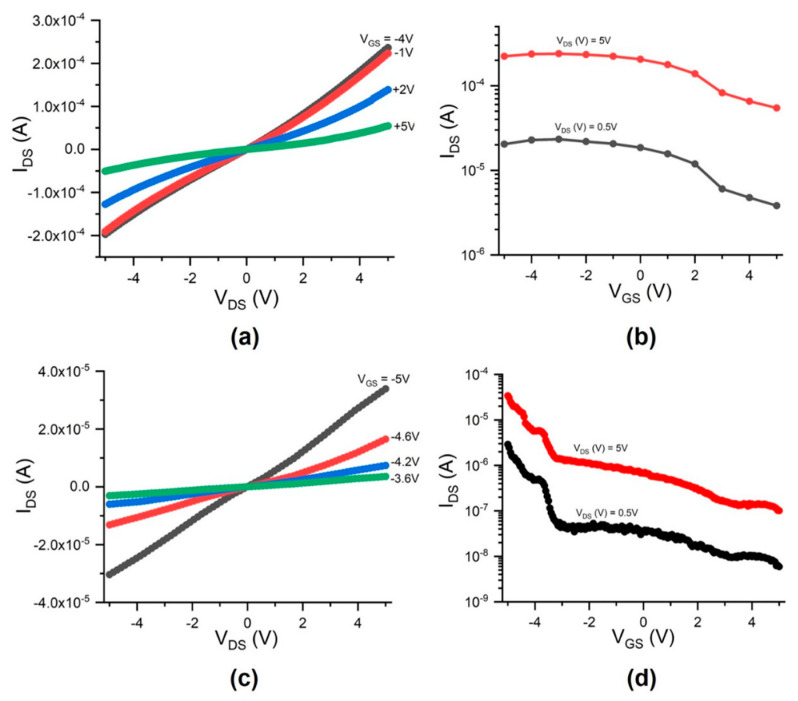
(**a**) Drain-source current (I_DS_) versus drain-source voltage (V_DS_) and gate voltage (V_GS_) and (**b**) transfer characteristics at V_DS_ = 5 V and 0.5 V for a functional CNTFET, respectively, where the I_DS_ on/off ratio is 4 at V_DS_ = 5 V and 6 at V_DS_ = 0.5 V; (**c**,**d**) Similar curves as in (**a**,**b**) but measured after the CNTFET was subjected to the electrical breakdown process, where the I_DS_ on/off ratio is 339 at V_DS_ = 5 V and 488 at V_DS_ = 0.5 V.

Figure 8a–d shows the comparison of the electrical characteristics of a fabricated CNTFET, which were measured as fabricated or after being subjected to the electrical breakdown process. Figure 8a,b shows the electrical characteristics of the CNTFET, which were measured as fabricated. Figure 8a shows the drain-source current (I_DS_) versus drain-source voltage (V_DS_) and gate voltage (V_GS_), while Figure 8b shows the transfer characteristics at V_DS_ = 5 V and 0.5 V, where the I_DS_ on/off ratio is 4 at V_DS_ = 5 V and 6 at V_DS_ = 0.5 V. Figure 8c,d shows the electrical characteristics of the CNTFET, which were measured after the CNTFET was subjected to the electrical breakdown process described in Section 2.4, where the I_DS_ on/off ratio is about 339 at V_DS_ = 5 V and 488 at V_DS_ = 0.5 V. Although it decreased the I_D_ values, the electrical breakdown process significantly increased the I_DS_ on/off ratios. This post-device fabrication process has been used to increase the I_DS_ on/off ratio of CNTFETs and decrease the leakage current [38,39]. In the CNTFET channel with the intermixed presence of metallic and semiconducting CNTs, the electrical breakdown process can selectively destroy, by Joule heating, the metallic CNTs responsible for the unwanted electrical characteristics while preserving semiconducting CNTs.

## 4. Summary

We present the wafer-scale fabrication of carbon nanotube field-effect transistors (CNTFETs) with carbon nanotubes as the active channel material by using the dielectrophoresis (DEP) process. A device wafer with 30 CNTFETs was fabricated and characterized successfully, and it was found that 87% of the fabricated CNTFETs was functional and that, among the functional CNTFETs, 30% of the CNTFETs had an I_DS_ on/off ratio larger than 20 while 70% of the CNTFETs had an I_DS_ on/off ratio under 20. The highest I_DS_ on/off ratio was 490. The variation of I_DS_ on/off ratios in the CNTFETs is mainly due to the effect of the metallic carbon nanotubes in the channel. If the source carbon nanotubes are pure semiconducting tubes or have fewer metallic tubes, the I_DS_ on/off ratios should be much higher and the variation of the ratios should be much smaller. The electrical breakdown was used to improve the I_D_ on/off ratios of the CNTFET, and it was found that it significantly increased the I_DS_ on/off ratios of the fabricated CNTFET. The DEP-based positioning of carbon nanotubes (CNTs) in the fabrication of CNTFETs is simple and effective. The DEP-based method can be easily extended for the assembly of other nanomaterials in the development of the wafer-scale fabrication of devices. The DEP-based device fabrication steps are compatible with Si technology processes, can be further optimized following process development protocols practiced by the semiconductor industry, and can lead to the wafer-scale fabrication of CNT electronic devices and sensors.

## Data Availability

The data presented in this study are available on request from the corresponding author.
